# Establishment and Validation of an Automated System for the Antifactor IIa Assay: A Case Study of Potency Assessment of a Pharmaceutical Gel Formulation

**DOI:** 10.1021/acsomega.4c00290

**Published:** 2024-04-13

**Authors:** Gokselin Ozgen, Merve Turk Gezer, Guliz Armagan, Petek Ballar Kirmizibayrak, Ayfer Yalcin, Ozgen Ozer, Banu Ozkirim Arslan, Gonul Kayar, Udaya Kumar Dude, Aysegul Kaymak Ozdemir

**Affiliations:** † Abdi İbrahim Pharmaceuticals Research & Development Center (R&D), Istanbul 34538, Türkiye; ‡ Faculty of Pharmacy, Department of Biochemistry, Ege University, Bornova, Izmir 35040, Türkiye; § Faculty of Pharmacy, Department of Pharmaceutical Technology, Ege University, Bornova, Izmir 35040, Türkiye

## Abstract

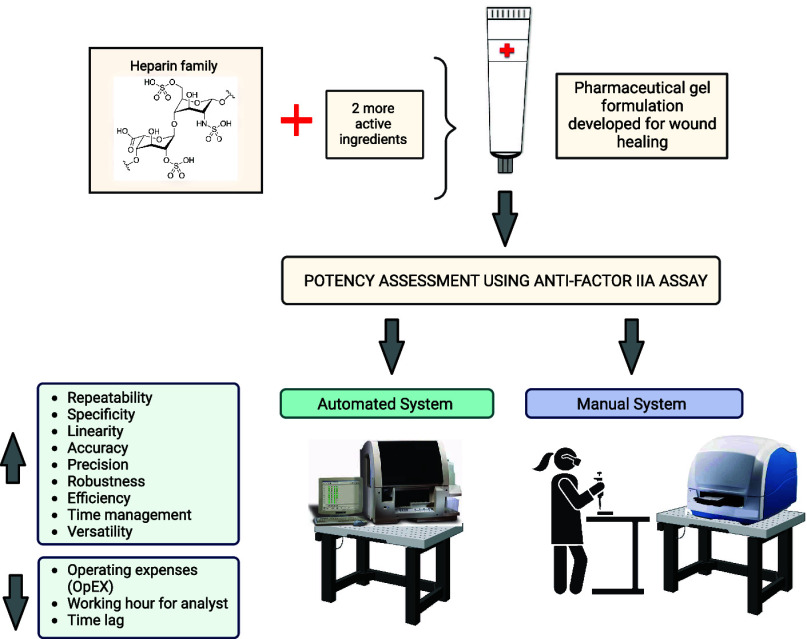

Antithrombotic agents and anticoagulant drugs, such as those from the heparin family, are employed in clinical settings for the prevention and treatment of clotting, thromboembolism, and wound healing. The potency assessment of antithrombotic agents is typically conducted using antifactor IIa assay with manual systems which are time-consuming and often lack repeatability. Here, we present a novel automated system that significantly enhances assay repeatability, attaining an outstandingly low relative standard deviation (RSD) % of only 0.6% for repeatability. This system has been applied to a pharmaceutical gel formulation for wound healing developed by Abdi Ibrahim Pharmaceuticals R&D Center as a case study for validation. The automated system demonstrated substantial improvements over manual systems in linearity (*R*^2^ = 0.9927), precision, accuracy, specificity, and robustness. The system aligns with the European Pharmacopoeia specifications, promising to enhance quality control across pharmaceutical formulations and conduct absorbance-based end-point assays within the pharmaceutical industry while offering increased throughput and cost-effectiveness.

## 1 Introduction

Antithrombotic agents and anticoagulant drugs, such as those within the heparin family encompassing enoxaparin, bemiparin, nadroparin, heparin, danaparoid, sulodexide, etc., are frequently employed to address complications arising from clotting and thromboembolism.^1^ The heparin family exerts its anticoagulant and antithrombotic effects by binding to antithrombin (AT), a serine protease inhibitor.^2^ Through this binding to AT, heparin effectively inactivates several serine proteases, including factor IXa, Xa, the TF-VIIa complex, and thrombin (factor IIa). Consequently, the laboratory determination of the biological activity of active ingredients within the heparin family often involves assessing this interaction with AT.^3,4^

**Figure 1:**
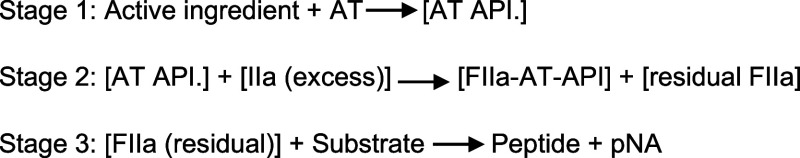
Working principle of the commercial kit (Biophen).

At the Abdi Ibrahim Pharmaceuticals R&D Center, a pharmaceutical gel formulation tailored for wound healing has been developed and manufactured. This formulation incorporates three active ingredients, one of which is part of the heparin family and is subject to confidentiality agreements. The determination of drug content in our pharmaceutical formulation relied on commercial product information and underwent analysis using the antifactor IIa assay for heparin as outlined in pharmacopeias.^5^ The end point of this assay was measured using a microplate reader following the guidelines in the European Pharmacopoeia (EP) monograph.^6^ To evaluate the potency of the active ingredient in the gel formulation, a commercially available kit (Biophen) was purchased. This kit includes exogenous antithrombin, thrombin, and a thrombin-specific chromogenic substrate. The operational principle of this kit (Figure 1) revolves around the inhibition of thrombin (factor IIa) by the tested active ingredient in the presence of exogenous antithrombin. As the thrombin-specific chromogenic substrate undergoes hydrolysis, paranitroaniline (pNA) is released, resulting in a light-yellow color. The quantity of released pNA correlates with the residual thrombin activity, and its absorbance is measured at 405 nm. Notably, there exists an inverse relationship between the concentration of the active ingredient and the intensity of the color obtained.^7^

While the 3-step reaction can be manually performed with the microplate reader, our group has innovatively introduced a new methodology by implementing a fully automated system using a coagulation analyzer compatible with a commercially available kit. After the repeatability parameter in the automated system was confirmed, analytical method validation was conducted following the ICH Q2 Validation of Analytical Procedures: Text and Methodology Guidelines for the relevant established method.^8^ The validation parameters included specificity, linearity, accuracy, precision, and robustness. Based on our data, we conclude that the analyses obtained with a manual system exhibit issues with repeatability. In contrast, analyses obtained with the automated system demonstrate significant advantages across various validation parameters, encompassing specificity, linearity, accuracy, precision, and robustness in addition to offering benefits in repeatability and time management.

To the best of our knowledge, this is the first study performing and comparing an antifactor IIa assay for heparin in a pharmaceutical gel formulation, utilizing both a manual system with a Thermo Scientific Varioskan Flash microplate reader and an automated system with the Instrumentation Laboratory ACL TOP 300 coagulation analyzer. Our established methodology not only proves effective for the antifactor IIa assay but also holds the potential to be employed as a valuable technique for conducting end-point assays based on absorbance within the pharmaceutical industry.

## 2 Materials and Methods

### 2.1 Materials

Human antithrombin (ATIII) (R1), purified human thrombin (R2), and chromogenic substrate specific for thrombin (CS-01(38)) (R3) were purchased from Hyphen-Biomed (France). European Pharmacopoeia reference standard was purchased from EP (Strasbourg Cedex, France). Tris(hydroxymethyl)aminomethane, polyethylene glycol 6000, hydrochloric acid, and acetic acid were purchased from Sigma-Aldrich (USA). Ethylenediaminetetraacetic acid (EDTA) and sodium chloride (NaCl) were purchased from Merck (Germany). All materials detailed above were of analytical grade.

### 2.2 Equipment

A Thermo Scientific Varioskan Flash microplate reader and an Instrumentation Laboratory ACL Top 300CTS coagulation analyzer were used in this study.

### 2.3 Solutions

Solutions and assay procedures are presented below for this study.

#### 2.3.1 pH 8.4 Buffer Solution

6.10 g of tris(hydroxymethyl)aminomethane, 2.80 g of EDTA, 10.20 g of NaCl, and 1.0 g of polyethylene glycol 6000 were weighed and dissolved in 1000 mL of purified water. pH was adjusted to 8.4 with hydrochloric acid. pH 8.4 buffer solution was used as the blank solution.

A commercially available kit was used for antifactor IIa activity. R1, R2, and R3 reagents were prepared as indicated below.

#### 2.3.2 R1 (ATIII (h)) Reagent

A vial of R1 was reconstituted in 1.0 mL of purified water and homogenized for 30 min at room temperature (18–25 °C) without foam formation. Just before use, 4.0 mL of pH 8.4 buffer solution was added and mixed. After being reconstituted, it can be stored at 2–8 °C for 15 days and at room temperature (18–25 °C) for 4 days.

#### 2.3.3 R2 (Purified Human Thrombin) Reagent

A vial of R2 was reconstituted in 1.0 mL of purified water and homogenized for 30 min at room temperature (18–25 °C) without foam formation. Just before use, 4.0 mL of pH 8.4 buffer solution was added and mixed. After being reconstituted, it can be stored at 2–8 °C for 15 days and at room temperature (18–25 °C) for 4 days.

#### 2.3.4 R3 (Chromogenic Substrate Specific for Thrombin (CS-01(38)))

A vial of R3 was reconstituted in 1.0 mL of purified water and homogenized for 30 min at room temperature (18–25 °C) without foam formation. Just before use, 4.0 mL of purified water was added and mixed. After being reconstituted, it can be stored at 2–8 °C for 15 days and at room temperature (18–25 °C) for 4 days.

#### 2.3.5 Standard Solutions

The entire contents of an ampule of the European Pharmacopoeia reference standard were diluted with pH 8.4 buffer solution to obtain a solution having a concentration of 100 IU/mL. Standard solutions were diluted to four concentrations (0.01, 0.015, 0.020, and 0.025 IU/mL) with pH 8.4 buffer solution.

#### 2.3.6 Test Solutions

Gel formulation containing the active ingredient was diluted with pH 8.4 buffer solution to obtain test solutions at concentration levels like those of standard solutions.

### 2.4 Procedure

#### 2.4.1 Antifactor IIa Assay

##### 2.4.1.1 Manual System with a Microplate Reader (End-Point Method)

This assay was analyzed by using a microplate reader, following the procedure below according to the kit. The design of the antifactor IIa assay is described in Table 1.

**Table 1:** Description of the Antifactor IIa Assay Using a Microplate Reader

	temperature and time	volume
sample (0.01, 0.02, 0.03, 0.04 IU/mL)	37 °C	40 μL
+R1 solution	37 °C, 2 min, incubate	40 μL
+R2 solution	37 °C, 2 min, incubate	40 μL
+R3 solution	37 °C, 2 min, incubate	40 μL
acetic acid solution (20%)	37 °C	80 μL

After the termination of the reaction with the addition of 80 μL of acetic acid solution (20%), each solution was pipetted into a 96-well plate. The absorbance of blank, standard, and test solutions was measured at a wavelength of 405 nm as the kit suggested by using a microplate reader. As recommended for the end-point method of the kit, the regression of the log absorbance against concentrations of standard and test solutions was calculated, and the potency of the substance examined was expressed as International Units of antifactor IIa activity per 100 g. All of the assay results were obtained at a confidence level of *p* = 0.95.

##### 2.4.1.2 Automated System with a Coagulation Analyzer

This assay was analyzed by using a coagulation analyzer following the procedure described below according to the kit. The design of the antifactor IIa assay is described in Table 2.

**Table 2:** Description of the Antifactor IIa Assay Using a Coagulation Analyzer

	temperature and time	volume
sample + R1 solution (0.01, 0.015, 0.02, 0.025 IU/mL)	37 °C, 2 min, incubate	40 μL
+R2 solution	37 °C, 2 min, incubate	40 μL
+R3 solution	37 °C, 2 min, incubate	40 μL

Blank, standard, and test solutions were transferred to the cuvettes, and the absorbance was measured at a wavelength of 405 nm as the kit suggested by using a coagulation analyzer. As recommended for the end point method in the kit, the regression of the log absorbance against concentrations of standard and test solutions was calculated, and the potency of the substance examined was expressed as International Units of antifactor IIa activity per 100 g. All the assay results were obtained at a confidence level of *p* = 0.95.

#### 2.4.2 Validation of the Antifactor IIa Assay Method with an Automated System

The parameters of the procedure were evaluated according to the ICH Q2 Validation of Analytical Procedures: Text and Methodology guideline which defines necessary parameters such as accuracy, precision, specificity, and robustness.^8,9^ In such studies, calculation of the lower limit of quantification (LLOQ) is not necessary as neither quantitative measurement nor purity assessment is performed. Moreover, the accuracy and sensitivity parameters are sufficient to establish the assay’s sensitivity, eliminating the need for further calculations. The performance specifications for the Varioskan FLASH plate reader (Thermo) are as follows: the accuracy is within ±2% or 0.003 Abs, whichever is larger, at 200–399 nm (0–2 Abs range); and within ±1% or 0.003 Abs, whichever is larger, at 400–1000 nm (0–3 Abs range). The precision is defined as SD < 0.001 Abs or CV < 0.5%, whichever is larger, at 450 nm (0–3 Abs range).

##### 2.4.2.1 Specificity

Specificity testing was performed to determine the method’s ability to measure only the substances intended to be measured in the analyzed sample. For the specificity test, the absorbance values of the blank, placebo, standard, and test solutions were measured.

##### 2.4.2.2 Linearity

The European Pharmacopoeia reference standard for antifactor IIa was used to prove the linear response relationship, and the absorbance values of the solutions prepared at 5 different concentrations (0.010, 0.015, 0.020, 0.025, and 0.030 IU/mL) were measured. The calibration curves were constructed, and the linear regression analysis was calculated by the least-squares regression method.

##### 2.4.2.3 Precision

The precision of the method was determined by system precision, repeatability, and intermediate precision and was expressed as the relative standard deviation.

System Precision: The absorbance of each solution prepared from standard solutions of 0.01, 0.015, 0.020, and 0.025 IU/mL was measured 4 times. The absorbance values obtained from the solutions and the relative standard deviation (RSD %) between them were calculated as recommended in the European Medicines Agency (EMA) Guideline for Bioanalytical Method Validation.

Repeatability: The repeatability was examined by assaying six consecutive six samples of gel formulation on the same day (intraday) and under the same experimental conditions against standard.

Intermediate Precision: The assessment of intermediate precision involved the analysis of six consecutive samples of gel formulation against the standard on two separate days (interday), along with the analysis conducted by a different analyst in the same laboratory setting (between analysts).

##### 2.4.2.4 Accuracy

The accuracy of the method was evaluated by introducing active ingredient raw material into a placebo at concentrations of 0.010, 0.015, 0.020, 0.025, and 0.030 IU/mL. A total of 15 samples, with 3 samples at each concentration level, were prepared, and the absorbance of each solution was measured twice.

##### 2.4.2.5 Robustness

Standard and test solutions were prepared and analyzed at certain time intervals for at least 12 h by keeping the storage conditions (5 °C) constant, the obtained absorbance values were recorded, and the similarity % was calculated.

## 3 Results

### 3.1 Comparison of the Manual and Automated Systems for the Antifactor IIa Assay

During this research, the determination of the antifactor IIa assay involved the utilization of two distinct systems: the manual and automated systems. Within both systems, standard and sample solutions were formulated in four different doses. Subsequently, absorbance values were measured, and calculations were carried out based on these recorded readings. Adhering to ICH Q2 guidelines, the analysis was systematically conducted by the same analyst on different days throughout the analytical validation process.^8^ Additionally, another analyst performed the analysis on a separate day to ensure consistency and verify that similar results were achieved when the analysis was conducted by a different individual. Notably, variations in absorbance values were observed due to software disparities between the microplate reader and the coagulation analyzer. Within the manual system, both standard and test solutions were prepared at concentrations ranging from 0.01 to 0.04 IU/mL, a wider range than in the EP monograph^6^ because initial laboratory investigations revealed no significant difference among concentrations of 0.010, 0.015, 0.020, and 0.025 IU/mL for the manual system. Hence, the concentration range had to be extended to 0.040 IU/mL to achieve a standard curve with an acceptable correlation coefficient (*R*^2^) standard, which is set as ≥0.990.^10^ Absorbance values were then measured using the microplate reader, and the corresponding log absorbance values against these concentrations are detailed in Tables 3 and 4.

**Table 3:** Results of the Manual System for the Antifactor IIa Assay

potency of active ingredient						
concentration (IU/mL)	log absorbance for antifactor IIa assay					
	standard solutions			test solutions		
	first day	2nd day	different analyst	1st day	2nd day	different analyst
0.01	2.064	1.922	1.692	1.895	1.946	1.873
0.02	1.637	1.364	0.961	1.423	1.462	1.375
0.03	1.224	0.894	0.651	0.857	1.087	1.010
0.04	0.955	0.657	0.415	0.604	0.775	0.717

**Table 4:** Results of the Automated System for the Antifactor IIa Assay

potency of active ingredient						
concentration (IU/mL)	log absorbance for antifactor IIa assay					
	standard solutions			test solutions		
	first day	2nd day	different analyst	first day	2nd day	different analyst
0.010	3.108	3.095	3.104	3.115	3.088	3.105
0.015	2.996	3.013	2.992	3.036	3.017	3.021
0.020	2.867	2.910	2.877	2.893	2.925	2.893
0.025	2.769	2.835	2.781	2.798	2.832	2.785

Significant differences were observed in the log absorbance values measured for 4 different concentrations of the standard solution between the first and second days. For instance, the log absorbance value for a concentration of 0.04 IU/mL of standard solution was 0.955 on the first day, whereas it was 0.657 on the second day, signifying an inconsistency in the interday analysis of the same sample. The differences in log absorbance become evident when values are compared between different analysts. The log absorbance value for the same concentration of the standard sample by a different analyst on a separate day was 0.415 corresponding to 43% of the log absorbance value obtained on the first day. Interestingly, the variations in log absorbance values for the test solutions were observed to be less pronounced compared with those for the standard solutions. One plausible explanation for this phenomenon is that the standard solutions comprise pure active ingredients, unlike the test solutions, and the analytical method employed might interact with other substances present in the formulation. Regardless of this phenomenon, achieving consistent results with a microplate reader proved challenging due to variations introduced by human factors during various stages of the assay such as reagent addition, mixing, and incubation.

Having shown that the manual system exhibits repeatability problems and not matching the acceptable *R*^2^ values even in a wider concentration range than the EP monograph, we established an automated system compatible with the commercial kit. Following the encouraging outcomes of the initial laboratory tests, standard and test solutions were prepared at concentrations ranging from 0.01 to 0.025 IU/mL aligning with the range specified in the EP monograph (within 0.005 and 0.03 IU/mL) for both convenience and to reduce cost.^6^

**Figure 2:**
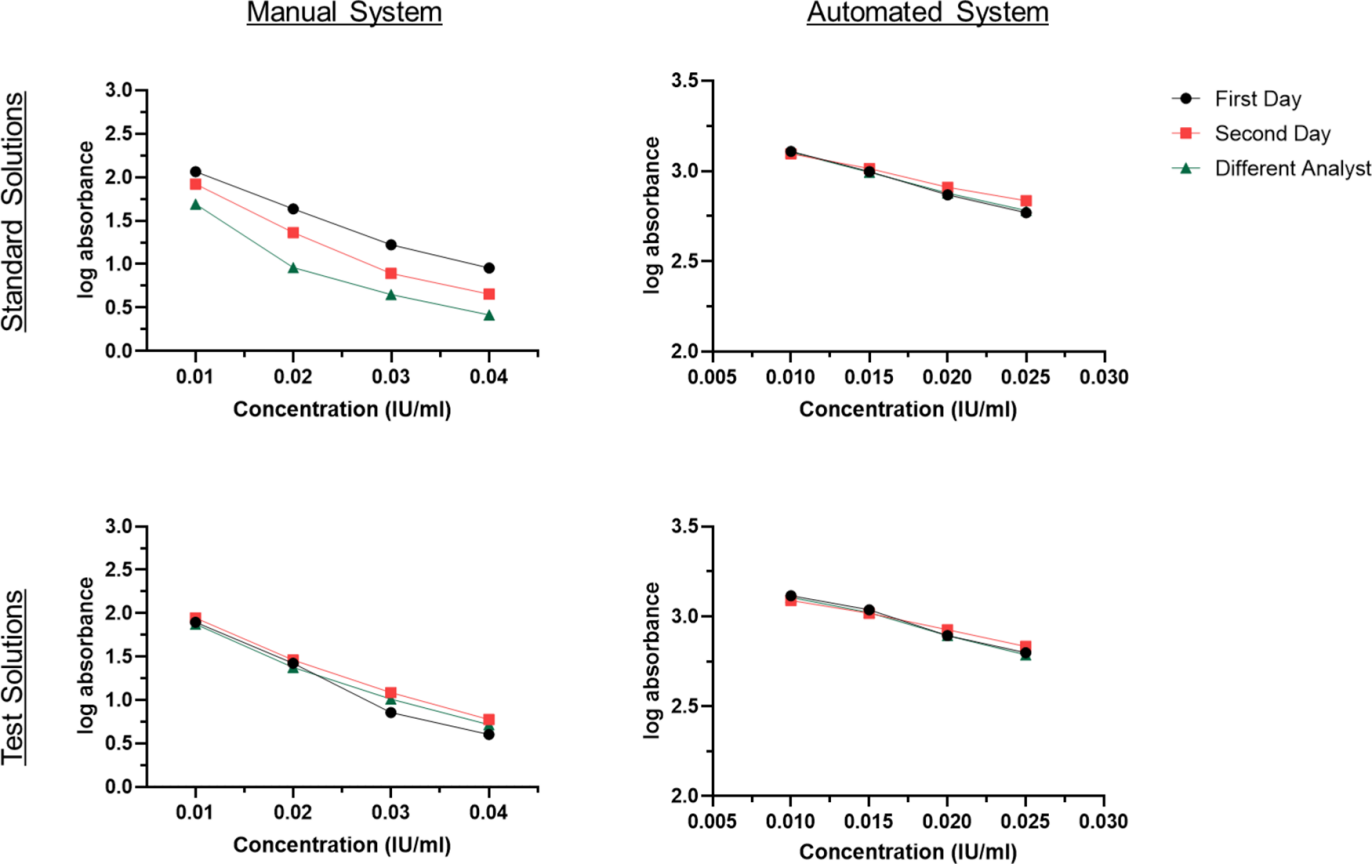
Comparison of the manual and automated systems for the antifactor IIa assay. Log absorbance values of four different concentrations of the standard and the test solutions measured by the same analyst on the first and second day and by different analysts on a separate day using the manual and automated systems. Absorbance values were then measured using a coagulation analyzer, and the corresponding log absorbance values against these concentrations are detailed in Table 4. Compared to the manual system, consistent log absorbance values were measured on different days by different analysts. For instance, the log absorbance value for a concentration of 0.015 IU/mL of standard solution was 2.996 on the 1st day and 3.013 on the 2nd day. More importantly, the log absorbance value for the same concentration of the standard sample by a different analyst on a separate day was 2.992.

Furthermore, when assessed through standard deviation, the difference in log absorbance values is 0.003 for the standard solutions and 0.01 for the test solutions, respectively, suggesting a greater precision and consistency in the measurements. For a more comprehensive comparison of the manual and automated systems, log absorbance results for corresponding concentrations, days, and different analysts are also presented in Figure 2. Collectively, the automated system yielded repeatable and precise results as these stages were executed by the device rather than individuals. Additionally, the device’s capacity to analyze several test solutions concurrently contributed to time efficiency.

Following the successful demonstration of the automated system addressing the repeatability issue present in the manual system for the antifactor IIa assay, further experiments were conducted on our pharmaceutic gel formulation to assess whether our established methodology could meet the validation parameters outlined in ICH Q2, encompassing linearity, precision, accuracy, specificity, and robustness.^8^

#### 3.1.1 Specificity

According to ICH Q2 guidelines, proving the specificity of a method requires avoiding the measurement of absorbance values from the active substance in blank and placebo solutions.^8^Table 5 provides log absorbance values plotted against concentration for specificity analysis.

**Table 5:** Specificity of the Antifactor IIa Assay Using the Automated System

solution name	absorbance (405 nm)	log absorbance
blank	not detected	
placebo	not detected	
standard solutions		
0.010 IU/mL	1337.26	3.126
0.015 IU/mL	1072.07	3.030
0.020 IU/mL	825.79	2.917
0.025 IU/mL	709.08	2.851
test solutions		
0.010 IU/mL	1380.18	3.140
0.015 IU/mL	1122.44	3.050
0.020 IU/mL	880.18	2.945
0.025 IU/mL	674.33	2.829

No absorbance was detected in the blank and placebo solutions confirming the specificity of the antifactor IIa assay method using a coagulation device for assessing the potency of the active substance.

#### 3.1.2 Linearity

**Figure 3:**
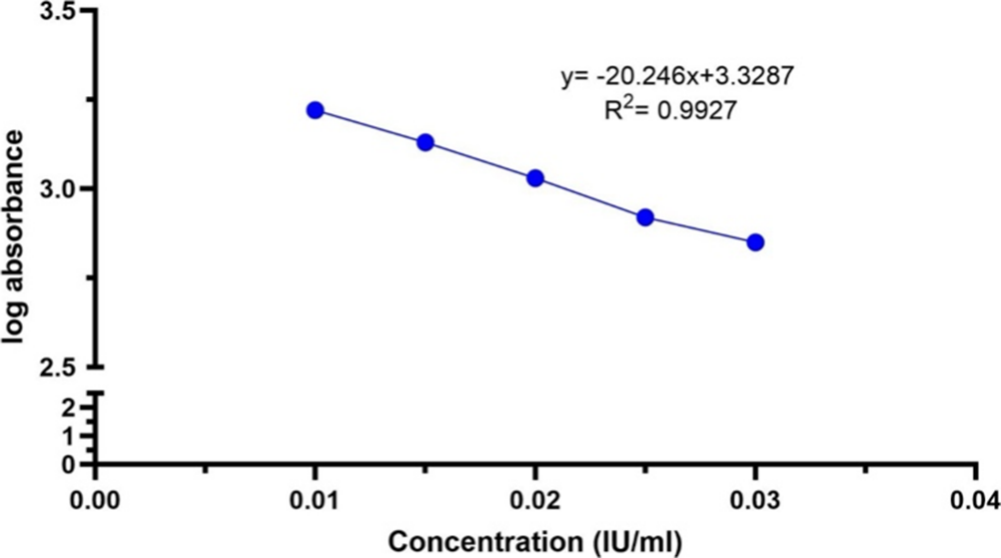
Linearity of the antifactor IIa assay using the automated system.

In order to evaluate the linearity of the calibration curve using the least-squares regression method, the absorbance values were acquired from a standard solution, formulated at five varying concentrations: 0.010, 0.015, 0.020, 0.025, and 0.030 IU/mL. Linear regression analysis yielded a correlation coefficient (*R*^2^) of 0.9927, indicating a value close to 1 (Figure 3), which signifies excellent linearity for the calibration curve in the automated system.

Moreover, a thorough analysis of variance (ANOVA) (Table 6) conducted on the acquired data demonstrates that the slope (*m*) of the graph falls within the range −22.66388 to −17.82884 at a 95% confidence interval. The exclusion of the value 0 within this range further confirms the linearity of the graph.

**Table 6:** Analysis of Variance

regression statistics	
multiple R	0.996321281
*R*^2^	0.992656095
adjusted *R*^2^	0.990820118
standard error	0.018212523
observations	6

ANOVA					
	df	SS	MS	*F*	significance *F*
regression	1	0.179337863	0.179337863	540.6693	2.02746 × 10^–5^
residual	4	0.001326784	0.000331696		
total	5	0.180664647			

	coefficients	standard error	*t* stat	*P-*value	lower 95%	upper 95%
intercept	3.328670325	0.016954937	196.3245509	4.04 × 10^–9^	3.281595874	3.375744776
X variable	–20.24636055	0.870725165	–23.25229747	2.03 × 10^–5^	–22.66388117	–17.82883993

#### 3.1.3 Precision

##### 3.1.3.1 System Precision

The precision of an analytical method indicates how closely a series of measurements, derived from repeated sampling of a uniform sample under specific conditions, agree with each other. Precision is evaluated at three levels: repeatability, intermediate precision, and repeatability. Typically, the precision of an analytical procedure is expressed as variance, standard deviation, or coefficient of variation, derived from a set of measurements.^8^ In this context, standard solutions were prepared at concentrations of 0.01, 0.015, 0.020, and 0.025 IU/mL, and the absorbance values were measured four times.

Table 7 presents the absorbance values and corresponding standard deviation values between absorbance measurements. RSD % values for the concentrations corresponding to 0.010, 0.015, 0.020, and 0.025 IU/mL were calculated as 2.8, 2.2, 5.6, and 1.9%, respectively. Collectively, these outcomes of the automated system not only meet the acceptable criteria for this method but also prove that the automated system is significantly more precise and sensitive as its RSD % values are significantly lower than the accepted threshold RSD % value which is 15% in the EMA Guideline.

**Table 7:** System Precision of the Antifactor IIa Assay Using the Automated System

concentration	0.010 IU/mL	0.015 IU/mL	0.020 IU/mL	0.025 IU/mL
1st absorbance	1282.90	1052.76	861.01	700.69
2nd absorbance	1345.94	1052.92	841.15	698.67
3rd absorbance	1358.53	1080.03	843.23	708.58
4th absorbance	1361.68	1102.57	757.78	728.37
average	1337.3	1072.1	825.8	709.1
SD^a^	36.9	24.0	46.2	13.6
RSD^b^ %	2.8	2.2	5.6	1.9
confıdence level (95%)	1337.3 ± 36.1	1072.1 ± 23.6	825.8 ± 45.3	709.1 ± 13.3

a SD, standard deviation.

b RSD, relative standard deviation.

##### 3.1.3.2 Repeatability

Repeatability indicates the degree of precision achieved under consistent operating conditions over a brief period. It is alternatively referred to as intra-assay precision. With this regard, six different test samples were prepared from the same pharmaceutical gel formulation of ours on the same day (intraday) and under the same experimental conditions. Then the target potency values were calculated compared to the standard as depicted in Table 8. The specified potency range for the evaluated gel formulation was set at 4500–5500 IU/mL, with an anticipated RSD% between results expected to be below 5%. Based on our measurements, the potency of our pharmaceutical gel formulation ranged from 4815 to 4892 IU/100 g, with an average potency of 4871 IU/100 g. The calculated relative standard deviation (RSD%) was 0.6%, significantly lower than the expected RSD% value. This further confirms the repeatability of the consecutive results from the six different test samples.

**Table 8:** Repeatability of the Antifactor IIa Assay Using the Automated System

potency			
test no	stated, IU/100 g	found, IU/100 g	confidence level (95%)
1	5000	4815	4550–5081
2	5000	4879	4579–5179
3	5000	4892	4416–5369
4	5000	4878	4549–5207
5	5000	4871	4563–5180
6	5000	4888	4693–5084
average		4871	
SD^a^		28.2	
RSD^b^ (%)		0.6	

a SD, standard deviation.

b RSD, relative standard deviation.

##### 3.1.3.3 Intermediate Precision

Intermediate precision reflects the extent of variability within a laboratory, encompassing variations across different days, analysts, equipment, and other relevant factors. In this context, six different test samples were prepared from the same pharmaceutical gel formulation of ours on two distinct days (interday) and subjected to analysis by a different analyst within the same laboratory (between analysts). The specified potency range for the evaluated gel formulation was set at 4500–5500 IU/mL, with an anticipated RSD% between results expected to be below 5%. The analyses conducted by the same analyst on different days (interday; Table 9) yielded a result of 4871 IU/100 g on the first day and 4766 IU/100 g on the second day. The calculated relative standard deviation (RSD%) between these results is 1.5%, which falls within the specified target values.

**Table 9:** Interday Precision Data of the Antifactor IIa Assay Using the Automated System

	potency			
day	stated, IU/100 g	found, IU/100 g	confidence level (95%)	RSD^a^ %
1	5000	4871	4848–4893	1.5
2	5000	4766	4543–4988	

a RSD, relative standard deviation.

The analysis result from the first analyst yielded 4871 IU/100 g, while the result from the second analyst yielded 5104 IU/100 g (Table 10). It is noteworthy that RSD% between the analysis conducted by different analysts is calculated as 2.6%, which falls within the targeted range. All together, these data underline the consistency and adherence of our automated system to the anticipated criteria.

**Table 10:** Between-Analyst Precision Data of the Antifactor IIa Assay Using the Automated System

potency				
analyst	stated, IU/100 g	found, IU/100 g	confidence level (95%)	RSD^a^ %
A	5000	4871	4848–4893	2.6
B	5000	5104	5050–5159	

a RSD, relative standard deviation.

#### 3.1.4 Accuracy

The accuracy of an analytical method signifies how closely the determined value aligns with an accepted reference value or a conventionally acknowledged true value. With this regard, placebo solutions were supplemented with active ingredients at concentrations of 0.010, 0.015, 0.020, 0.025, and 0.030 IU/mL, each administered three times, and then the accuracy was calculated (Table 11). According to the guidelines of the Food Drug Administration (FDA) and European Medicine Agency (EMA), the limit of accuracy should be within ±15.0% for pharmaceutical products.^9,11^ In our case, the RSD% for accuracy was found to be 5.6, which is significantly lower than the accepted RSD% value. Given all these data, it is evident that our automated system demonstrates superior accuracy for the antifactor IIa assay in pharmaceutical gel formulation.

**Table 11:** Accuracy of the Antifactor IIa Assay Using the Automated System

concentration (IU/mL)	test no	accuracy (%)
0.010	1	94.4
	2	93.2
	3	86.2
0.015	1	105.0
	2	98.7
	3	101.0
0.020	1	89.6
	2	95.7
	3	88.6
0.025	1	90.7
	2	93.5
	3	90.3
0.030	1	94.8
	2	90.8
	3	101.4
average		94.3
SD^a^		5.3
RSD^b^ (%)		5.6
confidence level (95%)		2.7

a SD, standard deviation.

b RSD, relative standard deviation.

#### 3.1.5 Robustness

The robustness of an analytical procedure is a measure of its capacity to remain unaffected by small but minor variations in method parameters, offering insight into its reliability during routine application. Hence, standard and test solutions were prepared and subjected to analysis at specific intervals over a duration of at least 12 h, maintaining constant storage conditions at 5 °C. Absorbance values for both standard and test solutions were recorded, with detailed outcomes in Table 12. According to the guidelines of FDA and EMA, the limit of stability should be within 85.0–115.0% for pharmaceutical products.^9,11^ The absorbance of the standard solution was consistently measured at regular intervals for a duration of 77 h to ascertain the repeatability of the system. The absorbance of test solutions was measured for 21 h as the prepared test solutions for each parameter were not stored at 5 °C for more than 21 h. As indicated by the robustness data, the standard solution exhibits stability for a duration of 77 h, while the test solution remains stable for 17 h under storage conditions at 5 °C. Notably, no significant variations were observed in the robustness results.

**Table 12:** Stability of Standard and Test Solutions under Storage Conditions at 5 °C

standard solution		test solution	
analysis time (h)	similarity (%)	analysis time (h)	similarity (%)
initial		initial	
21	105.8	21	102.1
27.5	102.0		
45.5	100.0		
51.5	102.3		
67	103.2		
77	103.8		

## 4 Discussion

The evaluation of antithrombotic potency in pharmaceutical gel formulations containing the heparin family involves the antifactor IIa assay with manual systems, known for its labor-intensive and time-consuming nature, albeit with less repeatability. In this context, an automated system based on the antifactor IIa assay was established to validate the antithrombotic potency of a pharmaceutical gel formulation developed by the Abdi İbrahim Pharmaceuticals R&D Center for applications in wound healing and containing a member of the heparin family as the active ingredient and is presented here as a case study.

The study revealed notable disparities in log absorbance values for reference standard solutions across different days and analysts, underscoring limitations for the antifactor IIa assay using the manual system, in particular for repeatability. Utilizing concentrations within the range specified in the European Pharmacopoeia monograph, the automated system exhibited improved precision and consistency compared with its manual counterpart. Standard deviation analysis corroborated the superior accuracy of log absorbance values obtained with the automated system, reinforcing its efficacy for antifactor IIa potency assessment in pharmaceutical gel formulations. Furthermore, the established automated system demonstrated highly favorable validation parameters including linearity (*R*^2^ = 0.9927), precision, accuracy, specificity, and robustness, in accordance with the ICH Q2 Validation of Analytical Procedures: Text and Methodology guideline. Notably, measurements conducted using the automated system indicated a target potency percentage within the concentration of 5000 IU/100 g in the evaluated gel formulation, ranging from 90.2 to 109.8%. This range aligns with the European Pharmacopoeia specification of 90–111%.

To gain a more comprehensive understanding of the validation parameters, we evaluated the performance of our automated system in executing antifactor IIa assays by benchmarking it against findings from four distinct validation studies. Our system showcased exceptional repeatability in a heparin derivative assay in a pharmaceutical gel formulation, with an RSD % as minimal as 0.6, outperforming the 1.04 to 1.18 RSD % ranges of other manual assays conducted in the solution form of heparin derivatives.^2,12^However, we observed some variations in other validation parameters. For instance, while the RSD % for repeatability in an enoxaparin sodium study was 1.18, the between-days and between-analyst precision RSD % were 0.45 and 1.01, respectively.^2^ Similarly, in a study on nadroparin calcium, RSD % for repeatability was reported as 1.04, and for interday and between-analyst precision as 1.65 and 1.32, respectively.^12^ It is noteworthy that the concentration range for both papers was not chosen in accordance with the US or EP monographs unlike our validation process.^5,6^ The differences in our system, however, can be attributed to a stricter concentration range adopted by the European Pharmacopeia and the complex nature of gel formulation assays. For instance, HPLC (high-performance liquid chromatography) validation for indomethacin and its degradation products in a topical gel reported precision RSD % values of 1.39, 3.25, and 3.10, underlining the challenges of gel assays.^13^ Compared to a validation study of the antifactor IIa assay of heparin adhering to the Japanese Pharmacopeia, where manual methods provided high accuracy and good precision in nine laboratories, our automated system maintained lower repeatability RSD % values.^4^ In addition, when compared to a fully automated laboratory robotic system (FA-LAS) used to analyze the cost and time involved in the drug development process, our accuracy rates were close, and our system exhibited superior repeatability.^4^ At this point, it should be underlined that, unlike the aforementioned paper, our method has not yet been fully automated, but despite this, it is similar to the fully automated system and even superior in terms of repeatability. This indicates that while the repeatability of our system is robust, precision and accuracy can be affected by the unique challenges of gel assays and the solid concentration range defined by the European Pharmacopoeia.

Finally, we conducted a thorough assessment of the pros and cons of each system, as detailed in Table 13. The automated system stands out in terms of precision, repeatability, and accuracy, and it offers several distinct advantages over the manual system. Though initially, there is a need to invest in a coagulation analyzer (CapEX: capital expenses), the ongoing operational costs (OpEX: operating expenses) are much lower with automation. This cost-efficiency comes from the decreased expenses on kits, standard reagents, and consumables, along with the reduced need for labor due to the system’s superior performance. In the validation phase, standards are prepared using a new vial each time because of stability considerations, with each vial costing €80. The standard curve must also fall within the required *R*^2^ value range. If the standards do not meet the *R*^2^ criteria, then they must be prepared again and again until the criteria are met to analyze the samples. There have been instances when we could not obtain the necessary *R*^2^ values, preventing us from analyzing any samples on the scheduled day and resulting in time lag for 24 h. This issue leads to not only the loss of materials but also disrupts the scheduling of experiments and consumes more time and labor for validation. Compared to the manual system, this reduces overall efficiency. When standards do meet the required *R*^2^ values, at most 40 measurements can be processed in the manual system within 3 h, which includes both preparation and measurement. In contrast, the automated system ensures a consistent attainment of *R*^2^ values, and it can measure 120 samples within the same duration. Its versatility is further demonstrated by its ability to perform other absorbance-based end-point assays, significantly extending its applicability. Our system is also compatible with a wide range of in vitro diagnostic tools, reagents, and data management systems, which are useful for diagnosing and managing thrombotic and bleeding conditions. For instance, when set up for activated partial thromboplastin time (APTT), it can conduct 110 tests per hour or 330 over 3 h. This marks a substantial increase in sample processing speed (or sample turn-around time) and efficiency, in addition to offering the advantage of more effective time management for experiments. In summary, the present automated system holds significant promise, not only for enhancing the quality control in pharmaceutical gel formulations but also for conducting end point assays based on absorbance in other pharmaceutical formulations in the pharmaceutical industry.

**Table 13:**
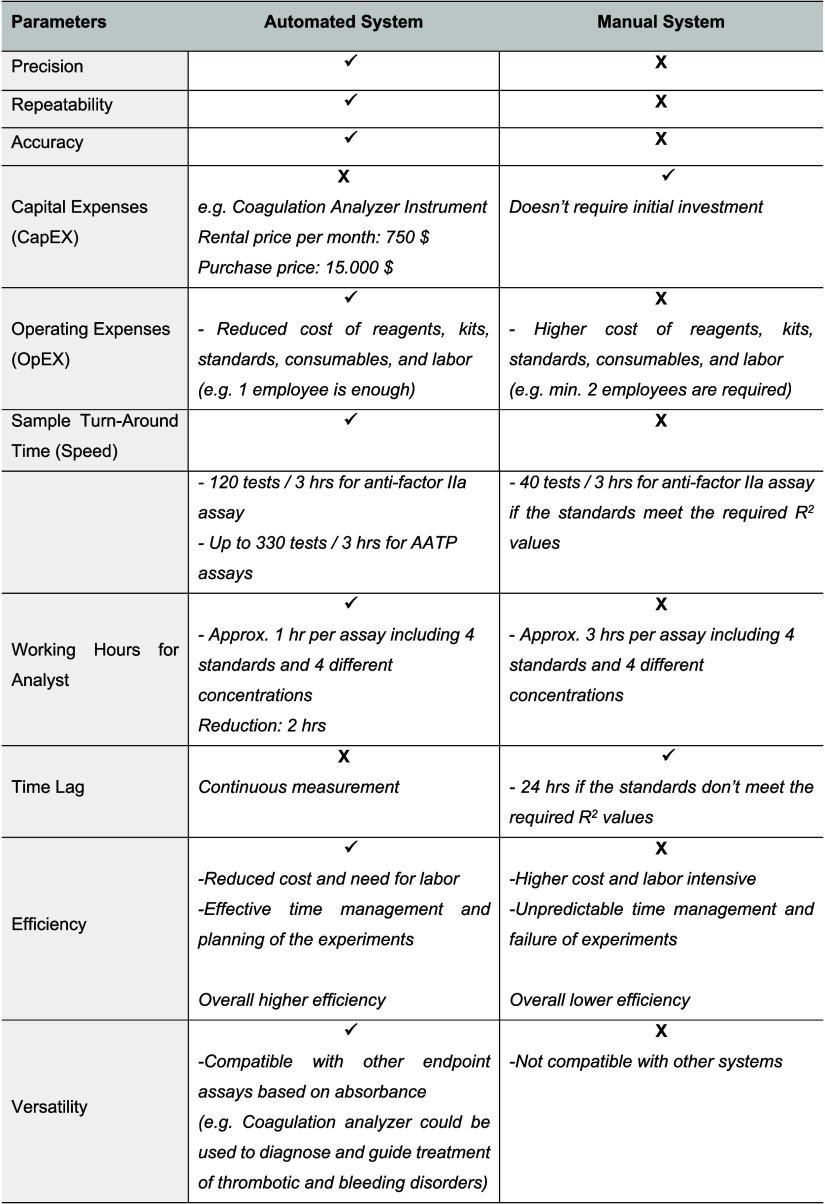
Comprehensive Comparison of the Automated and Manual Systems
